# Supplementary material for: Eastern oysters Crassostrea virginica settle near inlets in a lagoonal estuary: spatial and temporal distribution of recruitment in Mid-Atlantic Coastal Bays (Maryland, USA)
Source: PeerJ. 2023 Apr 27;11:e15114. doi: 10.7717/peerj.15114 (PMC10149057; doi:10.7717/peerj.15114)
Supplement: Supplemental Information 1 — Data about bottom type were obtained from the Mid-Atlantic Ocean Data Portal. [file peerj-11-15114-s001.docx]

| ID | Site name | Site type | Site location | Lat | Long |
| --- | --- | --- | --- | --- | --- |
| GC | Greys Creek | Pier | Tributary of Assawoman Bay | 38.45 | -75.12 |
| SM | St. Martin River | Bay | St. Martin River | 38.41 | -75.15 |
| TC | Turville Creek | Bay | Turville Creek | 38.36 | -75.15 |
| DP | DNR Pier | Pier | Sinepuxent Bay | 38.33 | -75.10 |
| VB | Verrazano Bridge | Bay | Sinepuxent Bay | 38.24 | -75.14 |
| IM | Island Mark 12 | Bay | Sinepuxent Bay | 38.22 | -75.17 |
| SP | South Point | Pier | Sinepuxent Bay | 38.22 | -75.19 |
| PL | Public Landing | Pier | Chincoteague Bay | 38.15 | -75.29 |
| TL | Taylor Landing | Pier | Chincoteague Bay | 38.08 | -75.36 |
| MI | Mills Island | Bay | Chincoteague Bay | 38.03 | -75.35 |
| GP | Guys Point | Pier | Chincoteague Bay | 38.01 | -75.39 |
| QS | Queen Sound | Bay | Chincoteague Bay | 37.92 | -75.40 |
| W | Wachapreague | Bay | Burtons and Bradford Bay | 37.61 | -75.69 |
|  |  |  |  |  |  |
