# Supplementary figures and images for: Eastern oysters Crassostrea virginica settle near inlets in a lagoonal estuary: spatial and temporal distribution of recruitment in Mid-Atlantic Coastal Bays (Maryland, USA)
Source: PeerJ. 2023 Apr 27;11:e15114. doi: 10.7717/peerj.15114 (PMC10149057; doi:10.7717/peerj.15114)

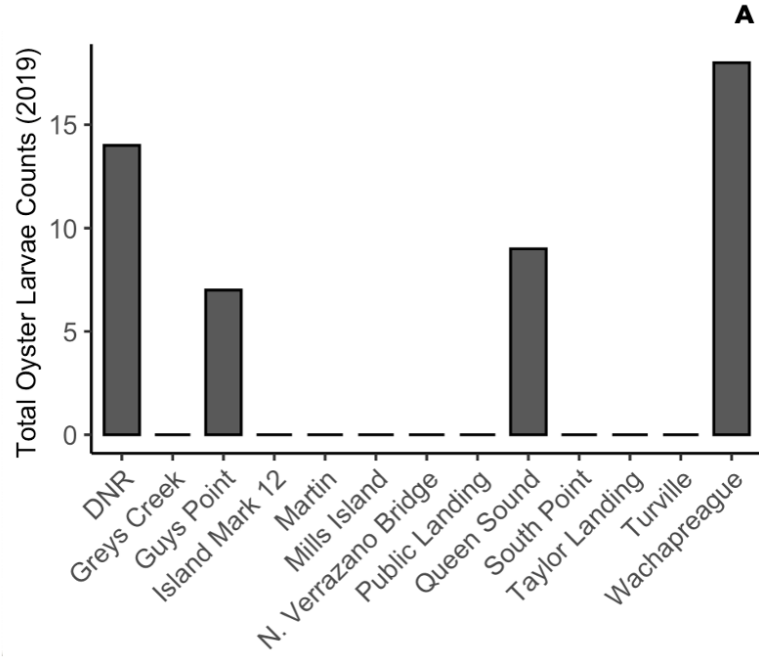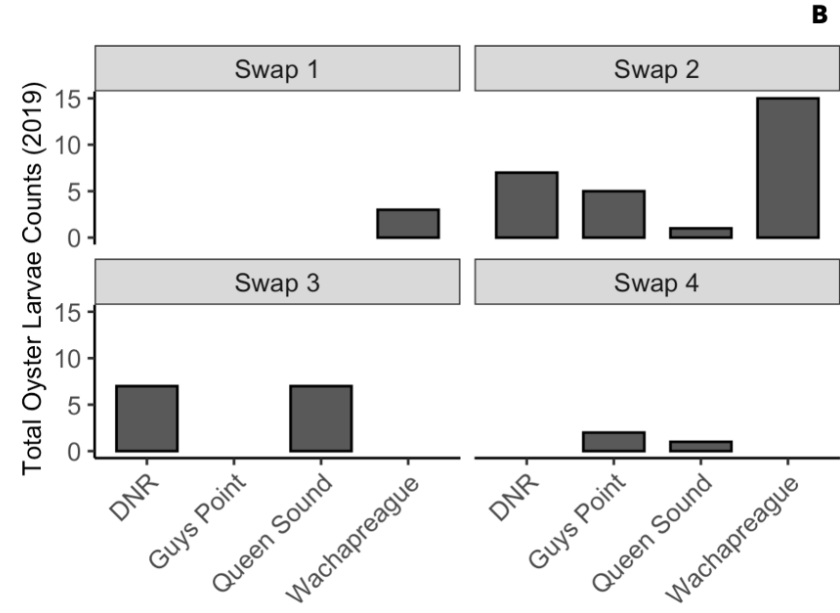

Supplement: Supplemental Information 2 — (A) Larval counts summed by site. All median larval counts were zeros during 2019 because there was only 15 observations total. (B) Total larval counts from the sites and facet wrapped by swap (sampling time point) when oyster larvae were observed during 2019. [file peerj-11-15114-s002.pdf]

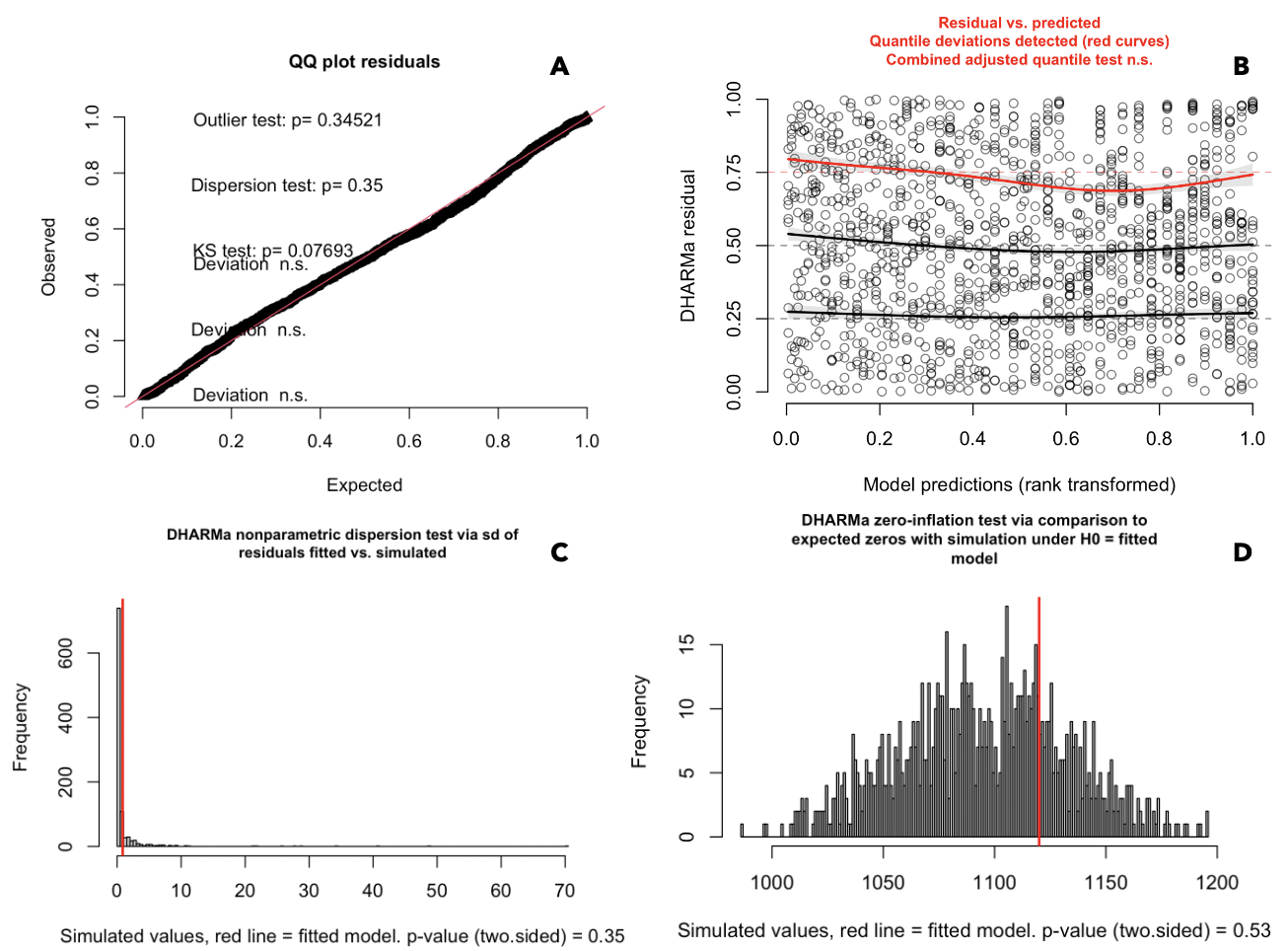

Supplement: Supplemental Information 3 — Model simulation of the residuals were run using the “simulateResiduals” function of the “DHARMa” R package with 1,000 simulations. (A) Results uniform quantile-quantile plot of the residuals and associated tests for uniformity with Kolmogorov-Smirnov (KS), over/under dispersion (dispersion test), and outliers. (B) Plot of the quantile regression of the residuals plotted against the model predictions. The red line indicates deviation from uniformity for the 0.75 quantile, but the combined quantile test was non-significant. (C) Shows expected distribution of zeros from the model simulated output against the observed values (ratioObsSim = 1.0212, p = 0.624). (D) Results of the simulation-based tests for over/under dispersion. [file peerj-11-15114-s003.png]

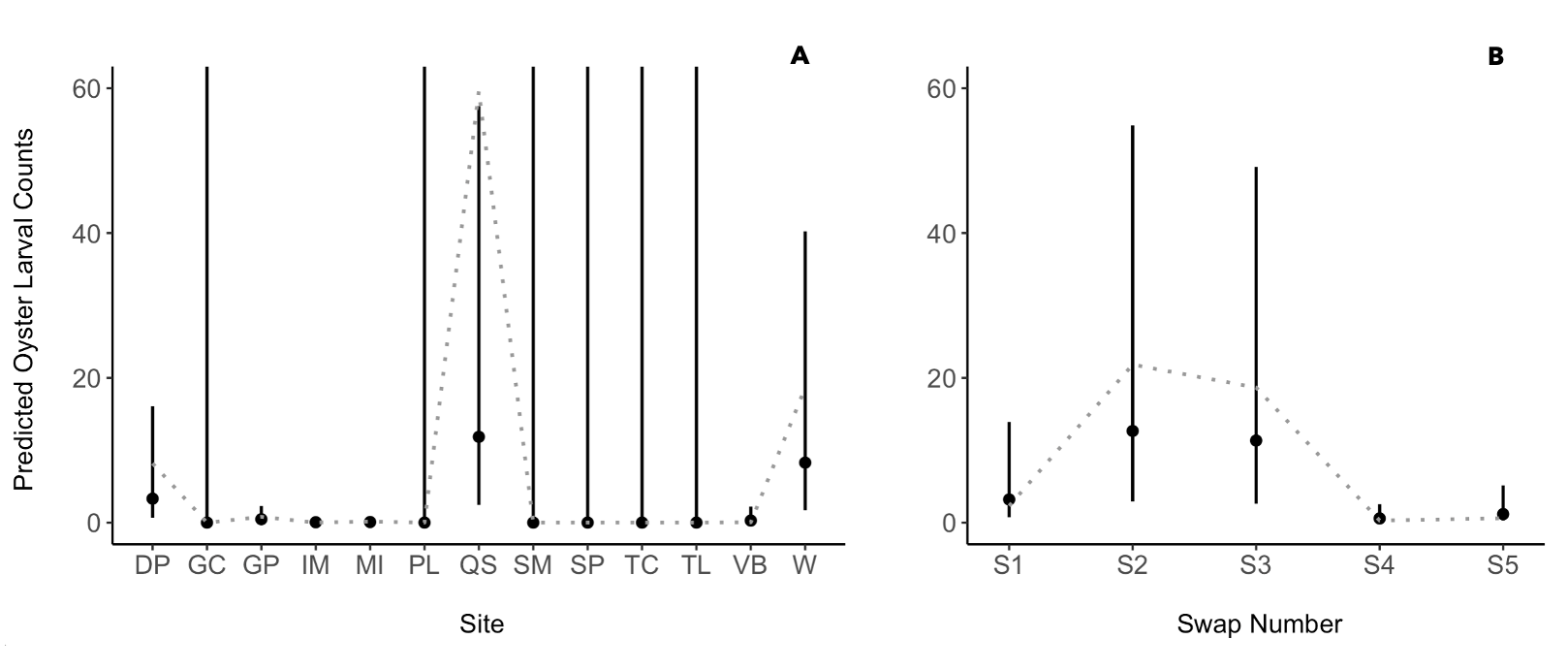

Supplement: Supplemental Information 4 — Results of the predicted oyster larval counts (points) with confidence intervals (vertical lines) from each (A) site (B) swap (sampling time points). The gray dotted line represents actual mean counts from the dataset. Model R code: glmmTMB(Larvae ~ Site + Sample Time + (1|Sampler Type) + (Level|Line number), ziformula = ~1, data = oy, family = poisson). [file peerj-11-15114-s004.png]

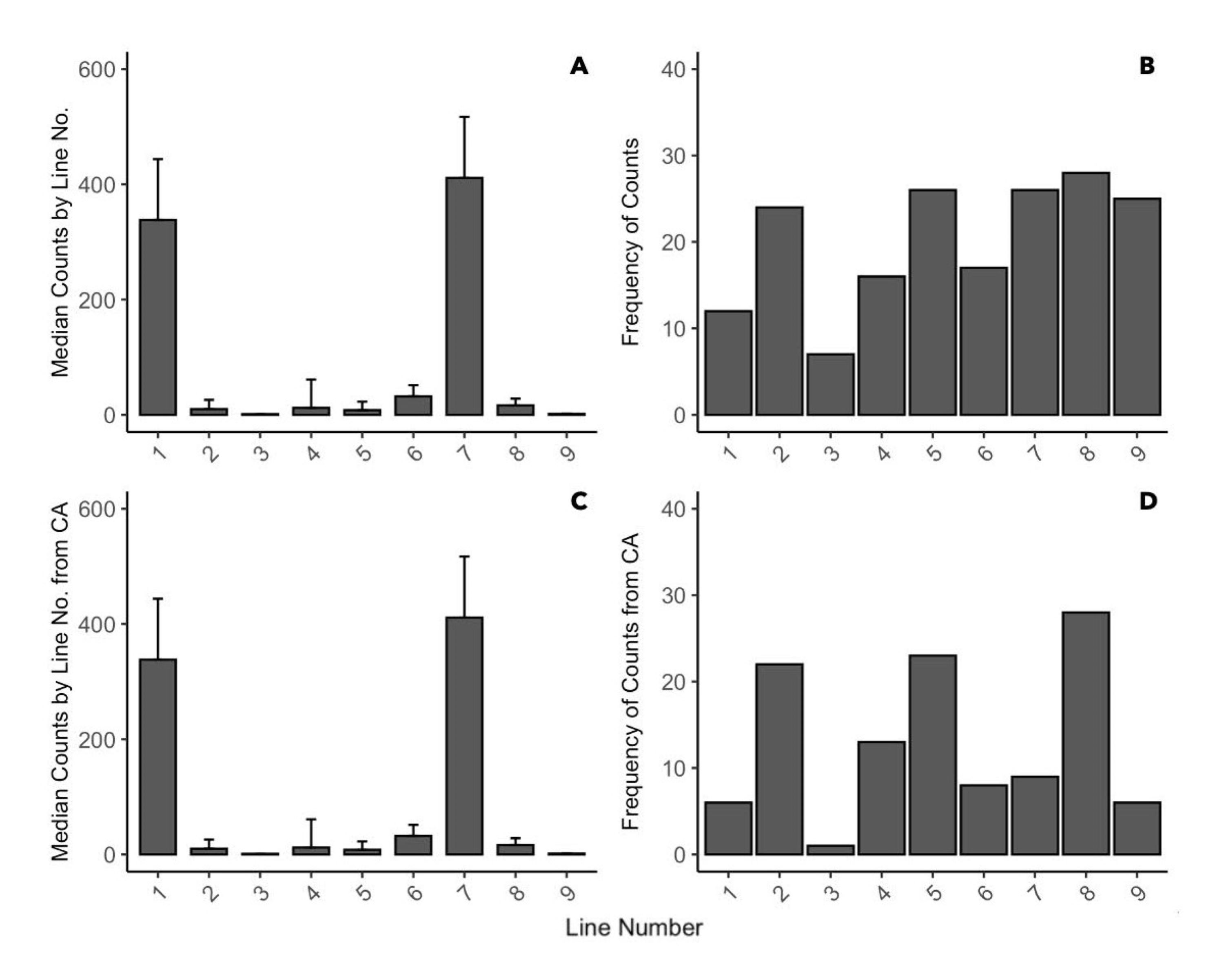

Supplement: Supplemental Information 5 — (A) Median larval counts from all three sampler types. (B) Frequency of larval observations from all three sampler types. (C) Median larval counts from ceramic arrays. (B) Frequency of larval observations from ceramic arrays. Data was pooled by sites and timepoints. [file peerj-11-15114-s005.jpg]

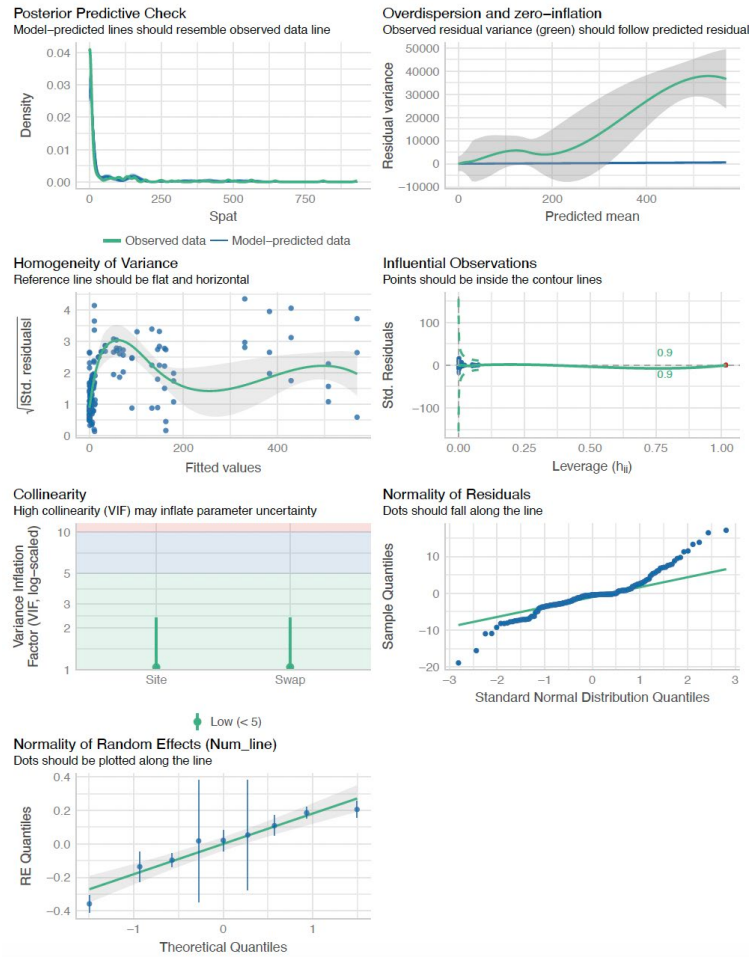

A

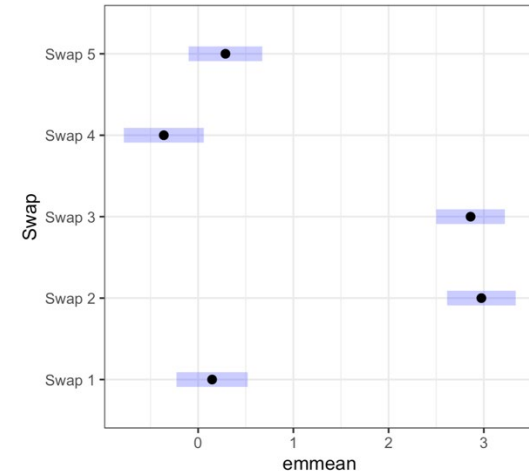

B

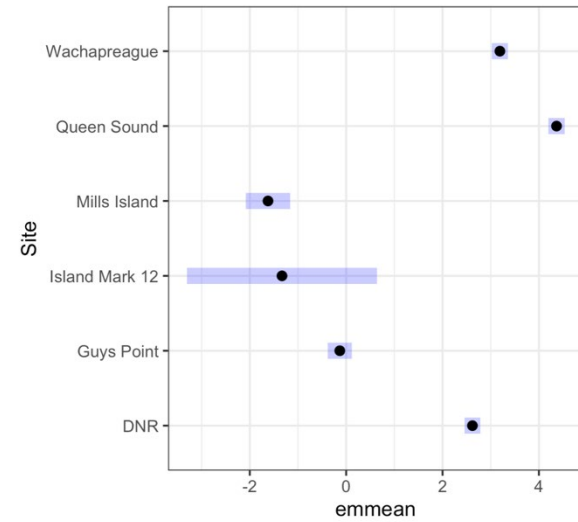

C

Supplement: Supplemental Information 6 — (A) Results from the quality check plots. (B) Larval count means from swaps derived from GLMM. (C) Larval count means from sites derived from GLMM. The model was generated using the lme4, easystats, and eemeans packages in R. Model contains a Poisson distribution with a log link function, site and swaps (time) fixed effects, and the position of the ceramic arrays (CA) on the pier or buoy as the random effect. No significant differences in larval counts occurred among plate positions on the ceramic arrays and resulted in zero variance as a random effect. The low observation numbers within sites and the percentage of zeros (42%) led to overdispersion/zero-inflation issues. Model R code: glmer(larval count ~ Site + Swap + (1|Num_line), data = oy20_CA, family = poisson (link = “log”), nAGQ = 0). [file peerj-11-15114-s006.pdf]
